# Breaking Bonds, Changing Habits: Understanding Health Behaviors during and after Marital Dissolution

**DOI:** 10.1177/00221465251320079

**Published:** 2025-03-04

**Authors:** Andrea M. Tilstra, Nicole Kapelle

**Affiliations:** 1 University of Oxford, Oxford, UK; 2 Trinity College Dublin, Dublin, Ireland; 3 Humboldt-Universität Zu Berlin, Dublin, Ireland

**Keywords:** divorce, health, life course, stress and coping

## Abstract

Marital dissolution is a stressful transition that can lead to unhealthy coping strategies, including smoking and drinking. Using fixed effect linear probability models to assess health behavior changes, we analyzed 6,607 women and 6,689 men in the Household, Income, and Labour Dynamics in Australia data set who were either continuously married or experienced marital separation between 2002 and 2020. We observed 1,376 separations (744 women, 632 men). We found that drinking and smoking increases leading to and in the year of separation, with variability by gender, education, and parenthood status. From Cox proportional hazards models, we showed that among individuals who smoked (N = 337) or drank (N = 756) in the year of separation, cessation was most likely for the highly educated and/or women. Unhealthy coping mechanisms throughout marital dissolution suggests a need for targeted support to those separating, especially for men and those with children and lower education.

Individual health and health behaviors build on one another throughout the life course (Halfon and Hochstein 2002). Literature has consistently pointed to social relationships as a site for understanding health behaviors because of both the network proliferation of health behaviors (De La Haye et al. 2011; Haas and Schaefer 2014) and the life course transitions often associated with them (Umberson, Crosnoe, and Reczek 2010; Umberson and Karas Montez 2010). Marital dissolution, as a major and disruptive life course transition, is thus related to changes in health behaviors (Leopold 2018; Umberson 1992). It is also important to consider the pathways between stressful life course transitions and health outcomes (Thoits 1983) because life course transitions—such as marital dissolution—are moments when behavioral change is common (Elder, Johnson, and Crosnoe 2003). The present study focuses on this link by examining health behavior changes over the marital dissolution process. We focus on alcohol consumption and smoking, two health behaviors that are precursors to later health and mortality (Lariscy, Hummer, and Rogers 2018; Pampel 2003).

The present article adds to the existing literature in four crucial ways. First, marital dissolution has often been measured as a single point in time event, ignoring the temporal dynamics and process nature of marital dissolution (for theoretical exceptions, see Amato 2000, 2010). Recent research emphasizes the processual nature of marital dissolution, encompassing the periods before, during, and after the event (Kapelle and Baxter 2021). Second, previous divorce research has often focused on health rather than health behaviors. Health behaviors, such as smoking and drinking, are critical because they are modifiable risk factors that directly impact health outcomes. We address these two gaps by using 20 years of annual panel data and a longitudinal approach that captures dynamic changes in self-reported drinking and smoking. This extends the work of Umberson (1992) and Leopold (2018), who focused only on immediate changes around marital dissolution. Third, research lacks insight into whether and how quickly individuals cease unhealthy coping behaviors after a stressful life event. A deeper understanding of the cessation of such behaviors is critical for a better understanding of when interventions are needed after stressful life events. Fourth, it is important to understand how individuals differ in their unhealthy behaviors around marital dissolution and the potential cessation. Among other aspects, coping abilities and mechanisms have been shown to differ for women and men but are also influenced by individuals’ educational level or parenthood status (Dykstra and Fokkema 2007; Harding, Whittingham, and McGannon 2021).

To address these gaps, the present article answers four research questions:

*Research Question 1:* How do health behaviors—specifically, drinking and smoking—change over the marital dissolution process?
*Research Question 2:* Do changes differ by gender, parenthood status, and education?
*Research Question 3:* For individuals who display adverse health behaviors in the year of separation, what is the likelihood of cessation in the years after separation?
*Research Question 4:* Which individual factors—focusing on gender, education, and the presence of children before separation—are associated with the cessation of adverse health behaviors in years after marital dissolution?

We use data from the Household, Income and Labour Dynamics in Australia (HILDA) survey for years 2002 to 2022. Australia provides an interesting context with high divorce rates and an overall rather low rate of smoking due to comprehensive tobacco control policies. At the same time, Australia has a relatively high prevalence of alcohol consumption, including problematic behavior such as binge drinking.

For our analyses, we rely on a total of 6,607 women and 6,689 men, including 1,376 marital dissolution transitions, to document changes in smoking and drinking over the marital dissolution process. We focus on marital separation as the trigger event in our analyses because it is the split of the joint household into two independent households, whereas divorce—the formal ending of a marriage—may or may not occur for sample respondents in the following years. Although separation from unmarried cohabitation is also a significant change to the life course, we focus explicitly on separation from marital unions. Our analyses proceed in two stages. First, we use fixed effects linear probability models to examine changes in smoking and drinking behavior over time, starting in years before marital dissolution until eight or more years after. Second, we use Cox proportional hazards models to explore the likelihood of smoking and drinking cessation in the years following marital dissolution—for respondents who displayed unhealthy behaviors in the year of marital separation. For both analyses, we identify differences by gender, education, and parenthood status.

The results shown here are important for identifying possible points of intervention from public health and medical professionals to support people throughout the marital dissolution process. Identifying the heterogeneities therein will further identify which individuals are at greater risk for engaging in poor health behaviors. The high divorce rates in Australia and Western societies more generally suggest that this will remain a relevant policy issue for years to come.

## Background

### Defining Health Behaviors and Their Relevance

Health behaviors are actions that either directly or indirectly affect individuals’ health and mortality (Armstrong 2009; Short and Mollborn 2015). Common examples of adverse health behaviors include poor diet and exercise routine, smoking, and substance use and abuse, all behaviors that increase the risk of premature death (Ezzati and Lopez 2003; Rogers et al. 2000). Smoking and drinking can also lead to serious later life health outcomes, including elevated risk of cancers and cardiometabolic health conditions (Lariscy et al. 2018; Pampel 2003). In Australia, tobacco smoking was the leading cause of preventable mortality in 2015 and was estimated to cost over 137 billion Australian dollars per year in tangible and indirect costs (Australian Institute of Health and Welfare 2020).

By themselves, health behaviors reflect an individual’s agency in the confines of their built environment (Armstrong 2009; Short and Mollborn 2015). Social relationships, at the meso-level of social life, are imperative for understanding health behaviors because they can encourage healthy or unhealthy behaviors (Umberson et al. 2010; Umberson and Karas Montez 2010). Research has highlighted that healthy behavior of peers is positively associated with one’s own behavior (Adams et al. 2022; Pruckner, Schober, and Zocher 2020). A highly influential social network is that of a romantic relationship. Support between partners and aspirations of a joint future together can help overcome unhealthy behaviors and may lead to more healthy behavior choices. As such, previous research has suggested that the transition into marriage is related to a reduction in risky health behaviors (Bachman 2002; Umberson et al. 2010). In turn, marital dissolution should also be considered as an important site for understanding how health behaviors might change as the advantages of the marital bond are lost and are combined with other stressors.

### Theoretical Framework: Stress and Health Behaviors

The life course is comprised of a series of developmental trajectories, connected by pivotal turning points or transitions, ranging from the transition to adulthood to the death of a close family member or the end of a relationship (Amato 2000; Lorenz et al. 2006). Disruptive life course transitions are more stressful because they deviate from normed expectations (Aneshensel 2015; Pearlin 2010). Depending on whether adjustment demands can be met or exceed personal coping resources (e.g., personality, network), stress levels associated with the transition or trigger event can vary (Thoits 2010; Umberson and Reczek 2007). Failure to cope, however, can lead to critical adverse consequences (Lazarus and Folkman 1984).

In moments of major stress, individuals adjust their health behaviors to cope (Pearlin 1999; Pearlin et al. 2005; Thoits 2010). For example, higher levels of stress from job loss or difficult legal situations are associated with higher quantity of alcohol consumption (Dawson, Grant, and Ruan 2005) and a higher likelihood of smoking initiation and continuity (Golden and Perreira 2015; Marcus 2014). Although marital dissolution can be a welcome transition if it reflects freedom from an unsatisfying or abusive relationship (Hawkins and Booth 2005; Schoen et al. 2002), the end of marriage is commonly considered stressful and emotionally demanding (Amato 2000). As outlined in Amato’s (2000) divorce stress adjustment model, the stressors experienced during separation can then result in poor emotional, behavioral, or health outcomes, indicating that the coping and management of stress during the marital dissolution process does not always happen healthfully.

Research has suggested that married people have better health than unmarried people, an effect that is stronger for men (Rendall et al. 2011; Umberson 1987). The end of a marriage is associated with higher levels of depressive symptoms and lower life satisfaction for the individuals experiencing the transition (Hewitt, Turrell, and Giskes 2012; Kalmijn 2017; Lorenz et al. 2006; Osborne, Berger, and Magnuson 2012) and even an elevated risk of death for men (Sbarra, Law, and Portley 2011). Existing research has also shown how health outcomes—including mortality and self-assessed health—change during marital dissolution (Leopold 2018; Shor et al. 2012; Williams and Umberson 2004).

### Health Behavior across the Marital Dissolution Process

Stressful experiences can proliferate through time (Aneshensel 2015; Pearlin 1999; Pearlin et al. 1981, 2005). The experience of a primary stressor and the precedent and subsequent changes to one’s life require major adjustments. Thus, marital dissolution—a disruptive transition in the life course and a primary stressor—likely results in changes to health behaviors beyond the time of transition. In line with recent divorce research (Kapelle and Baxter 2021), we broadly consider three stages of marital dissolution and theorize how health behaviors may vary across them. The three stages include (a) the anticipation of the separation, (b) the year of separation and immediate time after, and (c) the midterm to long-term aftermath.

The anticipation of marital separation is itself stressful because conflict between partners generally increases while marital satisfaction declines (Lavner and Bradbury 2010). In this process, spouses may grow apart, and emotional support between spouses declines. Because spouses are an important source of connection and support, feelings of loneliness increase prior to the actual separation (Kapelle and Monden 2024). We suspect that this may trigger unhealthy coping, including increased alcohol consumption or smoking.

The year of separation and immediate time after is commonly characterized by high emotional turmoil and a rise in secondary stressors, including financial challenges and network restructuring (McManus and DiPrete 2001; Raley and Sweeney 2020). For instance, separation is marked by spouses moving apart to establish two separate households. In that process, joint resources must be split in anticipation of legal divorce (Kapelle and Baxter 2021). The division of assets and divorce are often processes filled with conflict and financial losses. If dependent children were present, separation also heralds the transition to coparenting or single parenting (Adamsons and Pasley 2013; Osborne et al. 2012), which can lead to further conflict between ex-partners and increase financial stress, particularly for the custodial parent—commonly the mother. Separation regularly leads to substantial social network restructuring because family and friends may take sides and cut ties with one or even both partners (Terhell, Broese Van Groenou, and Van Tilburg 2004). This further exacerbates negative emotions and reduces the previously available support network that is essential for healthy coping, and we expect this to also result in elevated rates of poor health behaviors.

In the midterm to long term, individuals may start to overcome negative emotions associated with their relationship breakdown and improve their financial and social standings. This could include (re)establishing financial independence and adjusting living standards. From a social network perspective, individuals may rebuild or establish new social connections. This includes reentering the dating market and finding a new partner. As such, adverse coping mechanisms may transition into more positive ways of living, increasing the likelihood that individuals start (re)adjusting their health behaviors. We thus suspect a return to baseline health behavior levels in the midterm to long term after marital separation.

We build on previous literature that has examined the relationship between health behaviors and marital dissolution. Umberson (1987) found evidence of a higher risk of substance use and abuse, including problem drinking, for divorced U.S. Americans, especially for men. This study only compared respondents who were married to those who were divorced or widowed and thus is limited by the lack of longitudinal analyses. In 1992, Umberson then published estimates from two waves of data and showed an increase in tobacco and alcohol consumption for U.S. Americans transitioning out of marriage, an effect that was 4 and 6 times greater for men than women, respectively. This research, however, considered both divorces and deaths jointly and thus might not entirely capture the behavioral modifications that occur throughout the marital dissolution process. More recently, Leopold (2018) estimated an increase in smoking at the time of separation for German men and women, an effect that remains elevated for men but not women until three to five years after separation, the maximum period considered in the study. The study also found no change in drinking for women and a small increase for men. Although Leopold (2018) did use panel data, only three and seven biennial waves of data were available for drinking and smoking, respectively, and might not adequately capture nuances in the health behavior at the exact year of separation.

### Heterogeneities

The experience of marital dissolution and corresponding changes to health behaviors likely do not occur uniformly across sociodemographic characteristics. Women generally cope with separation better than men. Separated women have larger support networks and are less likely to be socially or emotionally lonely than men (Dykstra and Fokkema 2007; Leopold 2018). Additionally, women are more likely to initiate separation than men and are thus more prepared for the pending transition (Hewitt, Western, and Baxter 2006; Kalmijn and Poortman 2005). Rates of smoking and drinking are higher among men than women in Australia (Australian Institute of Health and Welfare 2020), and we suspect that this may be amplified during separation because of poor access to social support among men. This is supported by research on marital transitions, which suggests that men will have worse health behaviors during and after a marital transition (Leopold 2018; Umberson 1987, 1992). Literature on gender differences in smoking cessation is mixed but suggests that women have a more difficult time with smoking cessation than men (Smith et al. 2016), and research on alcohol abuse disorders has suggested that women are more likely to relapse than men (Holzhauer, Cucciare, and Epstein 2020). However, given that women cope better with marital separation than men, we expect that previously documented gender differences in cessation will not hold here.

Gender is likely to intersect with other social characteristics, including parenthood status and education, to affect health behaviors during separation. In recent years, there has been a normalization of “wine mom” culture or a reliance on wine (or other alcohol) as a coping mechanism for parenting (Harding et al. 2021). Although most mothers refrain from drinking during the day, when parenting activities are more prominent, many save it for the evenings when their children are asleep (Hill and Mazurek 2024). Additionally, single mothers in Australia have higher rates of binge drinking than coupled mothers (Maloney et al. 2010). The normalization of the wine mom and the high rates of binge drinking among single mothers suggest that women with children might exhibit higher rates of alcohol consumption at the time of separation than their childless peers. Smoking decreases with the transition to parenthood, especially for mothers, due to targeted public health advertising to decrease smoking during pregnancy (Bérard, Zhao, and Sheehy 2016). Those who do smoke attempt to keep it from their children (e.g., by only smoking at work; Blackburn et al. 2005). During marital separation, we suspect that smoking is likely to increase more for those without children and for those not living with their children—in Australia, this is most likely to be fathers.

There are educational gradients in smoking and drinking in Australia whereby individuals with lower levels of education are more likely to partake in both (Australian Institute of Health and Welfare 2020). These baseline differences may be explained by theories of cultural health capital and fundamental cause theories (Link and Phelan 1995; Shim 2010), which posit that individuals with higher levels of education are better able to access the resources and knowledge necessary to live healthy lives. In times of stress, those with resources may be better equipped to cope, and we expect this to be the case for the relationship between education and health behaviors.

### The Australian Context

#### Marital separation and divorce

In 2020, Australia had a crude divorce rate of 1.9 divorces per 1,000 residents, which was slightly higher than the average rate of 1.6 across the European Union but lower than the 2.3 in the United States (Australian Bureau of Statistics 2022; Centers for Disease Control and Prevention 2023; Eurostat 2023). Inherited mainly from the British common law, the Family Law Act 1975 and the Family Law Rules 2004 are the binding Australian legal frameworks that deal with all matters around separation and divorce. In Australia, marital separation is clearly distinguished from legal divorce. Spouses can only file for legal divorce after living separately for a continuous period of not less than 12 months. Whereas “separation” refers to the breakdown of the partnership with a split of the joint household into two independent households, “divorce” refers to the formal ending of a marriage.

##### Smoking and drinking

Australia’s legal age to purchase alcohol and cigarettes is 18 or older. Although there are strict laws banning all advertisements encouraging smoking or the use of tobacco products, there is a less stringent Alcohol Beverages Advertising Code Scheme that encourages responsible marketing of alcoholic products. As such, the smoking rates in Australia are lower than in most European nations and the United States (Ritchie and Roser 2023). In 2019, the daily smoking rate among individuals 14 and over was estimated at 11%. Among individuals with low education, 26% smoked daily, whereas 5% of college-educated individuals smoked daily. Rates of daily smoking for men and women are comparable, at 12% and 10% in 2019, respectively (Australian Institute of Health and Welfare 2020). Alcohol consumption in Australia is comparable to European nations and the United States (Ritchie and Roser 2022). In 2019, about 17% of Australians over the age of 14 drank regularly. Rates were higher among men than women, at 24% and 9%, respectively. Among individuals with low education, 22% drank regularly, and 15% of college-educated individuals drank regularly (Australian Institute of Health and Welfare 2020).

## Data and Methods

### Data and Sample Selection

For our analysis, we used longitudinal data from the HILDA survey (Release 22, years 2001–2023; Summerfield et al. 2015). The HILDA survey is a large, multipurpose panel survey that is mostly representative of the Australian population. Since 2001, the survey has collected annual information from respondents ages 15 years and older in eligible households via face-to-face interviews—or via telephone where needed—and self-completed questionnaires.

Our initial analytical sample included individuals ages 18 years and older living in private households if they either experienced a marital dissolution or stayed continuously married. We focused on the first observed marital separation as the trigger event because it refers to the split of the joint household into two independent households, whereas divorce refers to the formal ending of a marriage. As such, separation can be considered the more severe and incisive event. Note that our sample respondents may have proceeded to legal divorce in the years after separation.^1^ Alternatively, respondents may have chosen to stay separated without legal divorce, or legal divorce may have not been observed yet in the panel. In the years following separation, respondents may enter new partnerships. We stopped following respondents if they experienced the dissolution of another marriage—either through separation/divorce or the death of the partner. We excluded observations with missing values on any of our main analytical variables, which was the case for fewer than 2% of our sample. The final analytical sample was comprised of 6,607 women with 68,993 individual-year observations and 6,689 men with 67,746 individual-year observations. The sample included 1,376 separations, with 744 transitions for women and 632 transitions for men. This analytic sample was further restricted for the second part of our analyses, and this is described in the following. Table S.3 in the online version of the journal provides a descriptive overview of our initial sample, and Figure S.1 in the online version of the journal illustrates the sample selection process.

### Measuring Health Behaviors

We focused on two health behaviors: smoking and drinking. Both smoking and drinking behaviors were collected through the self-completed questionnaire, which was used for sensitive topics.

Smoking was assessed in the HILDA with the question, “Do you smoke cigarettes or any other tobacco products?,” with five response categories (see Table S1 in the online version of the journal). We collapsed responses into a binary indicator for *smoking* (0 = not at all, 1 = yes). Additionally, we generated a variable that captured *daily*—and thus heavier—*smoking* (0 = no daily smoking, 1 = daily smoking). The dichotomization was necessary to avoid potential biases in our analyses, computational challenges, and difficulties in interpretation. It is also a commonly used approach in panel data analyses for ordered variables.

A binary indicator for regular drinking (0 = no, 1 = yes) was generated based on the information for the questions “Do you drink” with originally eight response categories (see Table S1 in the online version of the journal). We defined regular drinking as drinking daily, weekly, or monthly. The reference category was thus “not drinking at all” or “drinking rarely.” We were also interested in *binge drinking*. To assess this, we relied on an additional question about the amount of alcohol normally consumed on days spent drinking (see Table S1 in the online version of the journal). To identify binge drinking, we used the definition from the Australian Health and Medical Research Council (National Health and Medical Research Council 2020), which classifies binge drinking as more than five standard drinks for women and more than seven standard drinks for men. We thus generated a binge drinking binary indicator (0 = no, 1 = yes) that differed for women and men.

### Analysis 1: Fixed Effects, Linear Probability Models

#### Explanatory measures and covariates

To explore how health behaviors develop over the marital dissolution process, we generated a categorical measure that captures the stage of separation (1) more than three years prior, (2) three years to one year prior, (3) the year of separation, (4) one to two years after, (5) three to four years after, (6) five to six years after, (7) seven to eight years after, and (8) more than eight years after separation. Continuously married respondents were added to the first category. This categorical measure provided relevant nuance to explore differences during times of anticipation, the separation year, and the immediate and midterm to long-term effects. At the same time, the grouping of years ensured sufficient cell sizes across the categories (for an overview of cell sizes, see Table S.2 in the online version of the journal). Table S.4 in the online version of the journal provides an overview of average levels of drinking and smoking by gender across the marital separation process categories. We additionally explored heterogeneities across gender (0 = male, 1 = female).

To further explore heterogeneities in health behaviors throughout the marital dissolution process, we ran two additional analyses. First, we stratified our analyses by education. For this, we generated a binary indicator for whether respondents achieved a university qualification prior to separation (0 = less than university degree, 1 = university degree). Second, we stratified by the presence of children in the household, which we operationalized as whether the respondent lived with their own child at any point immediately before the separation (0 = no children present, 1 = children present).

Regression models included only a small set of time-variant covariates because all time-constant measures were accounted for by our approach. Specifically, we adjusted for respondents’ age (categorical measure) to capture maturation effects, year indicators to adjust for underlying time trends, and a binary indicator for whether respondents reported being divorced after their initial separation. Because the association between marital dissolution and health behaviors can be expected to work partially through mechanisms such as repartnering, living arrangements, family support, or employment, we decided against accounting for those potentially mediating factors in our main analyses and keep our regression models parsimonious. An analytically appropriate analysis of such coping-relevant mechanisms is beyond the scope of the current article but should be considered for future research. However, because repartnering has been considered relevant in the divorce literature (Jansen, Mortelmans, and Snoeckx 2009; Lin et al. 2019), we ran supplementary analyses accounting for repartnership. Results for these analyses are in Figures S.2 to S.6 in the online version of the journal and are overall in line with our main results. In total, 545 respondents from our separation sample—just under 40%—repartnered in the years after separation. In line with previous research (Di Nallo 2019), repartnering was more common among men than women in our sample.

##### Methodological approach

To estimate changes in health behaviors, we used fixed effects linear probability models, using the pooled sample and separately for men and women, with a set of time-varying control variables. These models solely used within-individual variation while discarding any between-individual variation. As such, only characteristics that vary over time could enter the model, and all time-constant variables dropped out of the equation. As a result, all time-constant heterogeneity (observed and unobserved) was accounted for in the model. The fixed effects regression models were therefore ideal for assessing how health behaviors change as individuals experience a marital separation. We corrected standard errors for the clustering of observations within individuals. Additionally, we estimated fully interacted models to examine whether separation-related changes in the outcomes differed significantly between men and women. The replication code for all analyses is available at https://osf.io/tk5z2/.

### Analysis 2: Event History Models

#### Analytic sample

Our event history analyses explored the probability that a respondent engaging in an adverse health behavior at time of separation stops in the subsequent years. We again focused on smoking and drinking and ran analyses separately by health behavior. Thus, we further restricted the analytic sample to include only individuals who separated and who were engaging in the health behavior at the time of separation. In total, we observed 337 respondents (women = 171, men = 166) who smoked in the year of separation and 756 respondents (women = 368, men = 388) who drank regularly in the year of separation.

##### Methodological approach and covariates

We used Cox proportional hazards models to predict the likelihood of cessation of adverse health behavior after separation. Cox proportional hazards models have the advantage of allowing the underlying baseline hazard to differ at each time point (Allison 1982). For all individuals, entry into the model was centered on the moment of marital separation. Duration was then measured with a fine-grained, single-year measure calculated until the time of censor: cessation of the health behavior, exit out of the survey, or the end of the follow-up period, Wave 22 in the year 2023. Thus, duration ranged from 2 years for those who were censored the year after their separation to 20 years for those who were censored at the last possible observation after separation.

The baseline model, Model 1, estimated the baseline hazard of health behavior cessation for all in the analytic sample, controlling only for gender. Building in a stepwise fashion, Model 2 then adjusted for age at separation. Model 3 added education (coded as described previously). Finally, Model 4 added whether the respondent was living with their children prior to the separation (coded as described previously). Only results from Model 4 are presented in the main text; results from Models 1 to 3 and full tables for all four models can be found in the Appendix in the online version of the journal. Tests of proportionality indicated that health behavior cessation is proportional by gender and education, gender, and the presence of children. We conducted robustness analyses to assess how time-varying repartnership status affects our results, and results remained consistent with our main models (Tables S.11 and S.12 in the online version of the journal). About 40% to 45% of our event history samples repartnered at any point after separation. Repartnering was not linked to substantial drinking cessation rates but was linked to higher smoking cessation rates. Although it is beyond the scope of the current study, future research should further explore how other time-varying life course transitions in the years after separation are linked to the cessation of adverse health behaviors.

## Results

### Changes in Health Behaviors over the Marital Dissolution Process

**Figure 1.**
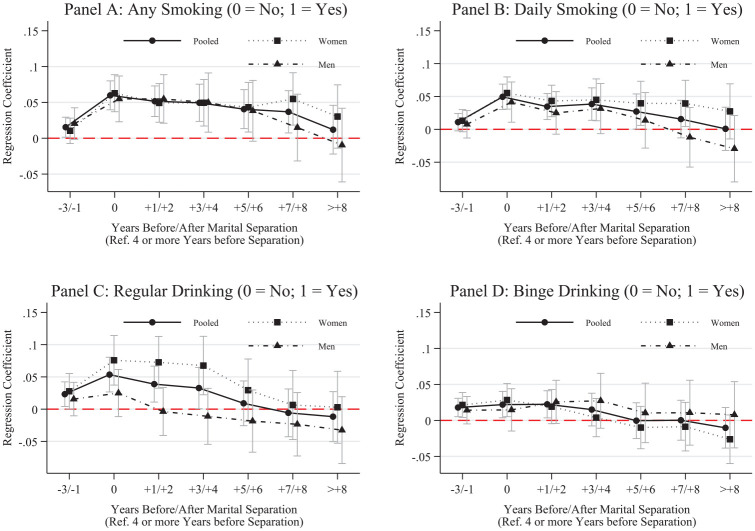
Fixed Effects Linear Probability Models for Smoking and Drinking. *Note:* Whiskers indicate 95% confidence intervals. N = 136,739 (women = 68,993, men = 67,746). Data are from the Household, Income and Labour Dynamics in Australia Survey (Release 22, years 2001–2023). Full model results are in Table S.5 in the online version of the journal. Predicted probability for the reference year is in Table S.6 in the online version of the journal.

We commenced our analyses with fixed effects linear probability models to explore changes in the probability of smoking or drinking over the marital dissolution process. Figure 1 shows pooled regression results (black lines) and the results disaggregated by gender (dotted line for women, dashed line for men).

Focusing on the pooled sample (Figure 1, round markers), we can see in Figure 1, Panel A that the probability of smoking substantially and significantly increased by 6% in the year of marital dissolution. The separation-related increases in the likelihood of smoking stayed elevated after separation, with minor declines after five years and an eventual return to predissolution levels after eight or more years. Additionally, our results highlight marginal, although statistically significant, anticipation effects, highlighting some health behavior changes in the lead-up to separation. Our results illustrate only marginal gender differences, with women increasing their likelihood of smoking marginally more than men in the year of separation and with slightly more elevated probabilities of smoking in seven and more years after separation compared to men. Because we compared changes to baseline levels of women and men, respectively, it is critical to note that baseline levels may already differ for women and men. At baseline, men were slightly more likely to smoke than women, with 14% compared to 10% smoking, respectively (Table S.6 in the online version of the journal). We show similar results for daily smoking in Figure 1, Panel B, although the increase is smaller for daily smoking than any smoking (i.e., daily and occasional). Additionally, men exhibited a slightly quicker return to the preseparation likelihood of smoking daily.

For drinking (Figure 1, Panel C), we found that the probability of regular drinking increased statistically in the year of marital dissolution by 5%. Over time, the probability of regular drinking declined for the pooled sample until it reached predissolution levels five or more years after separation. Similar to smoking, we found some anticipation effects, meaning that regular drinking increased prior to separation. Unlike smoking, we found substantial differences for women and men. However, women’s probability to drink regularly was substantially lower in the reference time frame (i.e., during marriage and at least four or more years before separation), when 52% of women and 72% of men drank regularly. Overall, women showed more substantial and lasting increases in their drinking compared to men. Men, in comparison, only marginally increased their drinking probability in the years before and in the year of separation, which returns to preseparation levels in the year after separation and declines even below reference levels in the following years.

In line with our results for smoking and regular drinking, results for binge drinking (Figure 1, Panel D) show increases in the likelihood of binge drinking before separation. Compared to the other measures, the probability of binge drinking did not increase further in the year of separation and declined over time to preseparation levels. Our results also show no substantial gender differences over the dissolution process and in our reference time frame, where 5% of women and 6% of men engaged in binge drinking.

### Heterogeneities by Education and Parental Status

**Figure 2.**
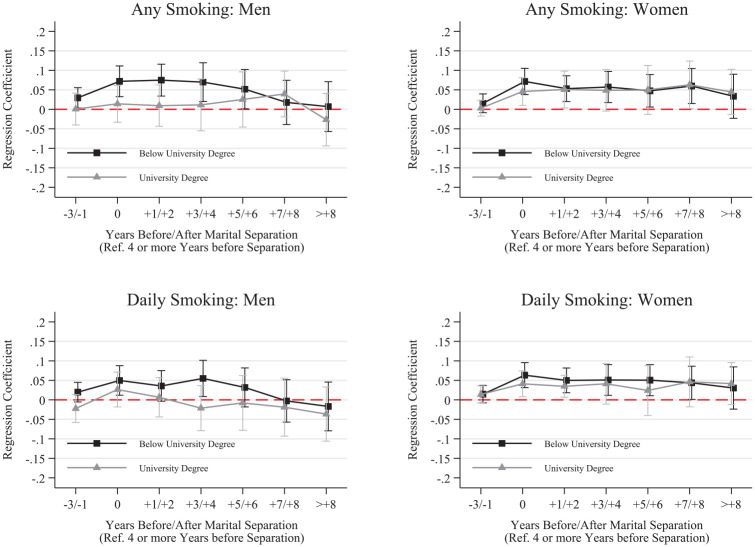
Fixed Effects Linear Probability Models for Smoking by Education. *Note:* Whiskers indicate 95% confidence intervals. N = 136,739 (women = 68,993, men = 67,746). Data are from the Household, Income and Labour Dynamics in Australia Survey (Release 22, years 2001–2023). Full model results are in Table S.7 in the online version of the journal.

**Figure 3.**
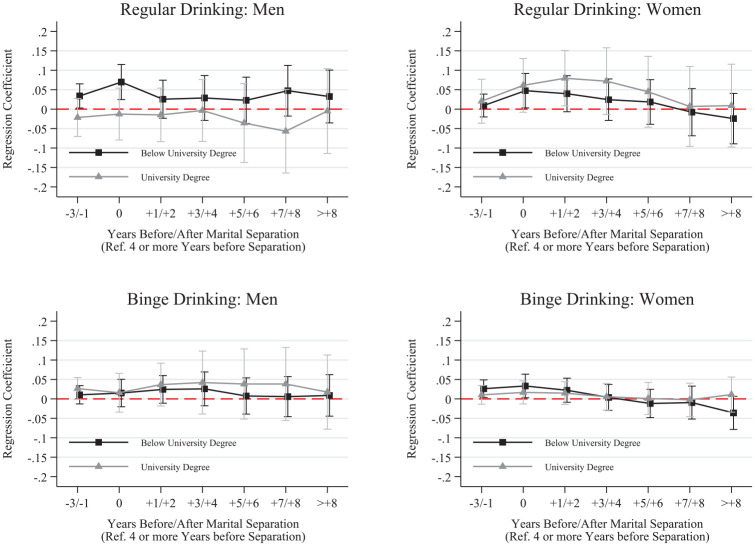
Fixed Effects Linear Probability Models for Drinking by Education. *Note:* Whiskers indicate 95% confidence intervals. N = 136,739 (women = 68,993, men = 67,746). Data are from the Household, Income and Labour Dynamics in Australia Survey (Release 22, years 2001–2023). Full model results are in Table S.8 in the online version of the journal.

**Figure 4.**
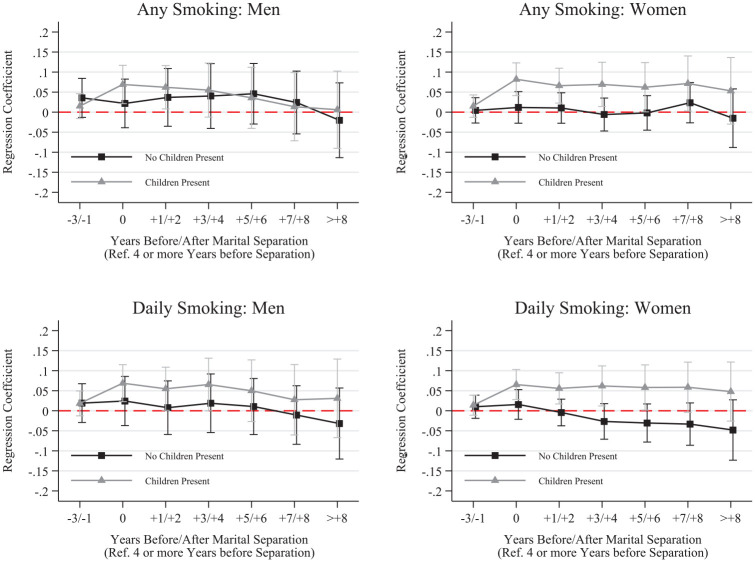
Fixed Effects Linear Probability Models for Smoking by Presence of Children before Separation. *Note:* Whiskers indicate 95% confidence intervals. N = 136,739 (women = 68,993, men = 67,746). Data are from the Household, Income and Labour Dynamics in Australia Survey (Release 22, years 2001–2023). Full model results are in Table S.9 in the online version of the journal.

**Figure 5.**
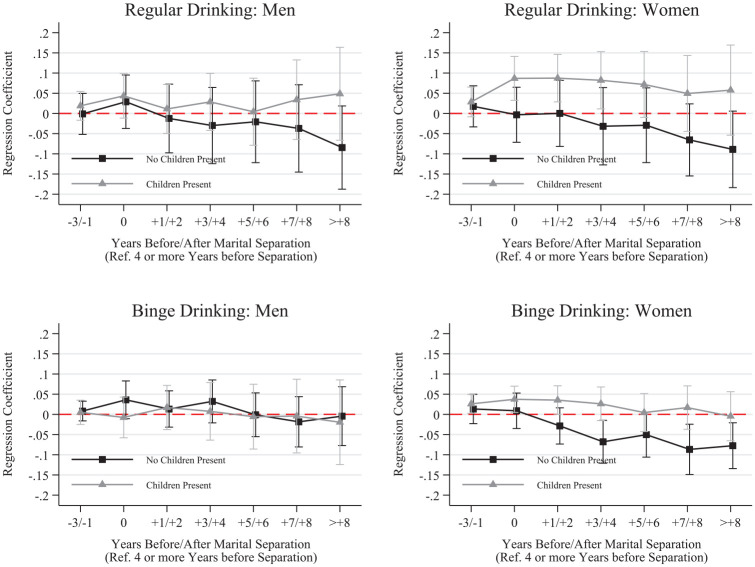
Fixed Effects Linear Probability Models for Drinking by Presence of Children before Separation. *Note:* Whiskers indicate 95% confidence intervals. N = 136,739 (women = 68,993, men = 67,746). Data are from the Household, Income and Labour Dynamics in Australia Survey (Release 22, years 2001–2023). Full model results are in Table S.10 in the online version of the journal.

We further disaggregated results by whether respondents completed a university degree before separation and whether respondents were living with their child immediately before separation. These results, shown in Figures 2 to 5, revealed several noteworthy patterns.

We saw little educational differences in smoking behaviors for women, but men with less than a university degree were more likely to start smoking during marital dissolution than men with a university degree, a pattern that remained elevated after separation (Figure 2). Similarly, highly educated men were more likely than less educated men to increase their regular drinking prior to and in the year of separation, and educational patterns for women were nearly identical (Figure 3). We found no substantial differences in men’s binge drinking likelihood by education.

Next, we focus on difference between those who lived with their child(ren) before separation and those who either did not or have no children. As shown in Figure 4, women and men who lived with their children before separation increased their likelihood of smoking and smoking daily in the year of separation, with lasting elevated levels the entire time after separation—except for men’s smoking likelihood, which declined to predissolution levels over time. For drinking across parenthood patterns, we do not see substantial differences in drinking behaviors except for regular drinking among women (Figure 5). Women who lived with their child(ren) immediately before separation increased their likelihood of being a regular drinker in the anticipation stage and in the year of separation. This probability remained elevated after separation. Conversely, the probability of regular drinking consistently declined among women not living with children before separation.

### Cessation of Adverse Health Behaviors in the Years after Marital Dissolution

**Figure 6.**
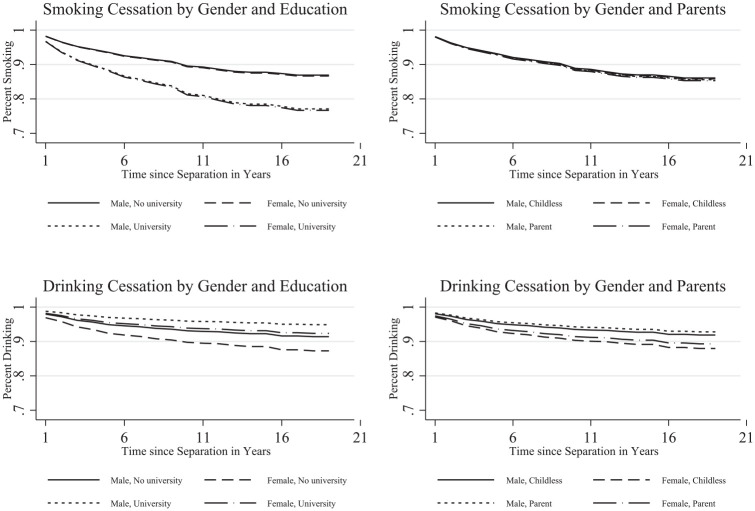
Event History Analyses to Explore the Cessation of Smoking and Drinking after Separation. *Note:* Smoking: N = 337 (women = 171, men = 166). Drinking: N = 756 (women = 368, men = 388). Data are from the Household, Income and Labour Dynamics in Australia Survey (Release 22, years 2001–2023). Full model results are in Tables S.11 and S.12 in the online version of the journal.

The final part of our analyses focused on the likelihood of smoking and drinking cessation after separation. For these analyses, we focused on respondents who smoked or drank regularly in the year of separation. We show results from Cox proportional hazards models in Figure 6, which illustrates the change in hazard rates for each gender by health behavior (top panels: smoking; bottom panels: drinking) and by social strata (left panels: education; right panels: presence of children). All panels were estimated at the means for all other covariates. Results highlight that among those who were engaged in the adverse health behavior at the time of separation, there was a greater likelihood of smoking cessation than drinking cessation.

Based on Model 1, women were 19% more likely to stop smoking after separation than men (hazard ratio [HR] = 1.19; Table S.11 in the online version of the journal). However, as additional variables are introduced, this effect diminishes, with only a 2% increased likelihood observed in Models 3 and 4, which is not statistically significant. Those with a university degree were 85% more likely to stop smoking after separation than those without a university degree. Lastly, living with children before separation does not significantly impact smoking cessation, with only a 4% increase in the likelihood of quitting, which is not statistically significant. These patterns were less pronounced with older age at separation. Specifically, with each additional year for the age at separation, the probability of smoking cessation reduced, highlighting the negative impact of age on the probability of quitting smoking (Table S.11 in the online version of the journal).

For drinking, gender is a significant predictor across all models. Women were 51% more likely to stop in the years after marital dissolution than men in Model 1 (HR = 1.51; Table S.12 in the online version of the journal). Contrary to results on smoking, having a university degree reduces the likelihood of quitting regular drinking. Individuals with a university degree are 42% less likely to quit drinking than those without a degree (HR = .58). Finally, living with children before separation does not significantly affect drinking cessation, with a 12% decrease in the likelihood of quitting observed in Model 4, which is not statistically significant. Results highlight that the greatest likelihood of drinking cessation occurred one to five years after marital separation. Akin to smoking, an older age at separation was associated with reduced odds of drinking cessation (Table S.12 in the online version of the journal).

## Discussion

Health and health behaviors build on one another throughout the life course (Halfon and Hochstein 2002), and stressful experiences, if not intervened on, have consequences for current and later health (Pearlin 1999; Pearlin et al. 2005; Thoits 2010). Although previous studies highlighted how marital separation and divorce are associated with adverse health outcomes (Shor et al. 2012; Williams and Umberson 2004), we build on this by examining health behaviors, a crucial pathway to health outcomes. By understanding how smoking and drinking change throughout the marital dissolution process, we shed light on possible points of intervention for public health and medical professionals seeking to improve individuals’ health.

Our work expands that of Umberson (1992) and Leopold (2018) by investigating how smoking and drinking changed throughout the marital dissolution process. By considering potential anticipation effects and up until eight and more years after separation, we acknowledged that marital dissolution is not a simple time-in-point event but should instead be considered as a dynamic process that is complex in its potential links to health behaviors. Our study uniquely explored changes in more critical and harmful behaviors by also focusing on daily smoking and binge drinking. Following respondents who smoked or drank in the year of separation, we then addressed the likelihood of cessation after separation. Finally, we explored heterogeneities in the health behaviors over the dissolution process and for the likelihood of cessation, looking at gender, education, and the presence of children prior to separation. To do this, we analyzed 6,697 Australian women and 6,689 men and used fixed effect linear probability models and Cox proportional hazards models.

First, we assessed how smoking and drinking evolved throughout the marital dissolution process. We found dynamic changes, suggesting that both smoking and drinking are coping mechanisms around marital separation. Specifically, we found that the prevalence of smoking and drinking increased at the time of separation for both women and men. What is especially noteworthy is women’s large increase in their probability to drink regularly, whereas men showed only marginal increases, suggesting links to the wine mom culture (Harding et al. 2021; Hill and Mazurek 2024). This pattern of elevated smoking and drinking dissipated over time, although only men fully returned to preseparation levels. The rising engagement in such coping behavior around marital separation is problematic for several reasons. Although we find that respondents mostly returned to preseparation smoking and drinking levels, even short spells of unhealthy coping behaviors can lead to long-term health consequences, including an elevated risk of death, and are financially costly (Lariscy et al. 2018). Additionally, separation may increase the risk of persistent smoking and drinking addiction and abuse, made further possible by the highly addictive nature of both substances (Australian Institute of Health and Welfare 2020).

Focusing on riskier health behaviors of smoking daily and binge drinking, our results showed marginal to moderate increases. The prevalence of daily smoking followed the pattern of “any smoking,” highlighting that the rising smoking prevalence around separation was not merely a result of smoking irregularly but of opting into highly adverse behaviors, such as smoking daily. By contrast, the binge drinking prevalence rose substantially less than the prevalence of regular drinking. Nevertheless, even small increases in the likelihood of binge drinking can have lasting health effects, possibly leading to alcohol dependency and abuse later in the life course (Kuntsche et al. 2017). Although women showed quick returns to preseparation binge drinking levels, levels stayed elevated longer for men, suggesting that men have more unhealthy coping than women.

Second, we explored how smoking and drinking patterns may differ by respondents’ education and the presence of children in the household before separation. Although our results showed no substantial educational differences over marital dissolution for women, we found some noteworthy differences for men. Men without a university education were more likely to increase their smoking and drinking compared to men with a university education. In fact, men with a university degree showed barely any changes in their smoking and regular drinking probabilities, suggesting better coping with the separation for those men compared to lower educated men.

Women who were living with their children before separation showed increases in their probability of smoking (at all and daily) and drinking regularly. The smoking probabilities stayed elevated throughout the study, and the probability of drinking regularly declined, although never fully reaching preseparation levels. These results are worrying but not surprising. Children commonly stay with their mothers during a separation, which means that women often carry the burden of single parenthood, including financial difficulties or balancing care and work responsibilities. Thus, women with children were likely to experience a range of additional stressors compared to women who did not live with children. Such high and lasting stress levels seemed to be linked to unhealthy coping that can potentially develop into persistent patterns. For men, we only found substantial differences for daily smoking. Men with children showed lasting increases in this health behavior, and men with no children showed only marginal changes in their daily smoking.

Third, we considered the likelihood of smoking and drinking cessation after marital separation. Smoking is highly stigmatized in Australia, which perhaps explains the nearly 20% cessation rate despite the highly addictive nature of nicotine. Those who did stop smoking after marital separation were likely to be highly educated and/or female. The accessibility of cultural health capital for highly educated people might lend itself to better resources on how to stop smoking (Shim 2010). The educational gradient to smoking in Australia coupled with its stigmatization may exaggerate these tools. The findings for women are interesting given that women usually have a more difficult time with smoking cessation (Smith et al. 2016). This counterintuitive finding could perhaps be explained by the greater social support that women experience throughout marital separation (Dykstra and Fokkema 2011).

The likelihood of drinking cessation is, on average, lower than smoking cessation, which may be tied to the cultural acceptability of drinking in Australia (Savic et al. 2016). Unlike smoking cessation, drinking cessation is greater for individuals with less than a university degree. This might be related to the high costs of marital dissolution and the high costs of alcohol prices in Australia, creating a financial strain that is felt more strongly by individuals from lower socioeconomic status backgrounds. Like smoking, the higher likelihood of drinking cessation among women might be because of their social networks. Additionally, childcare responsibilities most often fall to women during marital separation, which might further restrict women’s time and money to drink alcohol.

Our study has a few limitations. First, it is limited by its reliance on self-reported health behaviors. Comparing self-reported smoking and drinking behavior with more objective measures, such as biomarkers or peer-based observations, researchers have previously found some minor to moderate underreporting (Northcote and Livingston 2011). Such underreporting might be due to cognitive recall bias and sociocultural desirability bias. Additionally, previous research highlighted that problematic health behaviors—such as heavy drinking—tend to be underreported (Northcote and Livingston 2011). Thus, our results may be conservative estimates of changes in health behaviors, and true changes may be even more substantial. However, health behaviors in the HILDA survey were collected through a self-completed questionnaire, which might have resulted in less biased responses than those requiring answers to an interviewer. Irrespective of the actual level of bias introduced by self-reporting, our data are unique in measuring health behaviors and marital transitions over a 20-year time frame. No comparable data for objectively measured health behaviors are currently available. Second, we dichotomized measures for health behaviors because of computational challenges and bias that would likely be introduced by estimating panel data regressions with ordered categorical outcome measures. Although this dichotomization reduces the depth of information, it is a commonly used technique across research with similar methodological approaches. Third, due to sample size restrictions and the scope of the current article, we were not able to explore all potential mediators and moderators, although we encourage future research on this. Finally, we focus on dissolution of marital unions and do not consider separation from unmarried cohabitation. Rates of cohabitation are rising in Australia (Qu 2020), and future research should consider how this life course transition might also affect health behaviors.

Despite these limitations, our findings offer important contributions to a more thorough understanding of health behaviors during marital separation. The rising levels of smoking and drinking around marital separation point to critical coping behaviors with potential long-term health effects. With almost every second marriage predicted to end in separation, this life course transition is a critical event in Western societies, with health consequences across the population. The lower likelihood of drinking cessation after marital separation speaks to the broader culture around substance use in Australia. Smoking is a highly stigmatized health behavior, as seen in the stringent laws around tobacco and nicotine products. Drinking, however, is more socially accepted, and there is minimal guidance around alcohol advertising. The time leading up to, at, and shortly after marital separation is stressful, and if people turn to socially accepted behaviors (e.g., binge drinking) as their primary coping mechanism, this can have consequences for health and well-being. To combat this, Australia and Western societies more broadly might start discussions around more stringent alcohol laws, including restrictions on alcohol advertising, to minimize the social and cultural acceptability of excessive alcohol consumption. Life transitions, such as marital separation, are exceptionally stressful, and individuals undergoing this transition will need help and support for healthy coping to minimize adverse consequences for their current and later health.

## Supplemental Material

sj-docx-1-hsb-10.1177_00221465251320079 – Supplemental material for Breaking Bonds, Changing Habits: Understanding Health Behaviors during and after Marital Dissolution Supplemental material, sj-docx-1-hsb-10.1177_00221465251320079 for Breaking Bonds, Changing Habits: Understanding Health Behaviors during and after Marital Dissolution by Andrea M. Tilstra and Nicole Kapelle in Journal of Health and Social Behavior

sj-pdf-2-hsb-10.1177_00221465251320079 – Supplemental material for Breaking Bonds, Changing Habits: Understanding Health Behaviors during and after Marital Dissolution Supplemental material, sj-pdf-2-hsb-10.1177_00221465251320079 for Breaking Bonds, Changing Habits: Understanding Health Behaviors during and after Marital Dissolution by Andrea M. Tilstra and Nicole Kapelle in Journal of Health and Social Behavior

## Notes

1. In Australia, partners must be separated for 12 months before filing for divorce. Once filed, divorce proceedings typically take 4 to 6 months, but disputes over custody, property, or service can extend this to 1 to 2 years or more.
