# Supplementary material for: Breaking Bonds, Changing Habits: Understanding Health Behaviors during and after Marital Dissolution
Source: J Health Soc Behav. 2025 Mar 4;67(1):86–103. doi: 10.1177/00221465251320079 (PMC12936151; doi:10.1177/00221465251320079)
Supplement: sj-docx-1-hsb-10.1177_00221465251320079 – Supplemental material for Breaking Bonds, Changing Habits: Understanding Health Behaviors during and after Marital Dissolution [file sj-docx-1-hsb-10.1177_00221465251320079.docx]

**Journal** of **Health**

and **Social Behavior**

OFFICIAL JOURNAL OF THE AMERICAN SOCIOLOGICAL ASSOCIATION

**ONLINE SUPPLEMENT**

**to article in**

Journal of Health and Social Behavior

**Breaking Bonds, Changing Habits: Understanding Health Behaviors during and after Marital Dissolution**

**Andrea M. Tilstra**

*University of Oxford*

**Nicole Kapelle**

*Trinity College Dublin*
