# Supplementary material for: Breaking Bonds, Changing Habits: Understanding Health Behaviors during and after Marital Dissolution
Source: J Health Soc Behav. 2025 Mar 4;67(1):86–103. doi: 10.1177/00221465251320079 (PMC12936151; doi:10.1177/00221465251320079)
Supplement: sj-pdf-2-hsb-10.1177_00221465251320079 – Supplemental material for Breaking Bonds, Changing Habits: Understanding Health Behaviors during and after Marital Dissolution [file sj-pdf-2-hsb-10.1177_00221465251320079.pdf]

Supplementary Material

Table S.1 Information on the generation of dependent variables and original HILDA variables

Variables and coding	Original HILDA variables: Questions and response categories
Any smoking<br>0 No → categories 1 and 2<br>1 Yes → categories 3 to 5<br><br>*Daily smoking*<br>0 No → categories 1, 2, 4 and 5<br>1 Yes → category 3	"Do you smoke cigarettes or any other tobacco products?" [surveyed annually since wave 2]<br>1. No, I have never smoked<br>2. No, I no longer smoke<br>3. Yes, I smoke daily<br>4. Yes, I smoke at least weekly (but not daily)<br>5. Yes, I smoke less often than weekly
Regular drinking<br>0 No → categories 1, 2 and 8<br>1 Yes →categories 3 to 7	"Do you drink" [surveyed annually since wave 2]<br>1. No, I have never drunk alcohol<br>2. No, I no longer drink alcohol<br>3. Yes, I drink alcohol every day<br>4. Yes, I drink alcohol 5 or 6 days per week<br>5. Yes, I drink alcohol 3 or 4 days per week<br>6. Yes, I drink alcohol 1 or 2 days per week<br>7. Yes, I drink alcohol 2 or 3 days per month<br>8. Yes, but only rarely
Binge drinking<br>0 No → categories 6 and 7 for women, categories 5 to 7 for men<br>1 Yes → categories 1 to 5 for women, categories 1 to 4 for men	"On a day that you have an alcoholic drink, how many standard drinks do you usually have?" [surveyed annually since wave 2, only surveyed if the respondent indicates that they drink at all]<br>1. 13 or more standard drinks"<br>2. 11 to 12 standard drinks<br>3. 9 to 10 standard drinks<br>4. 7 to 8 standard drinks<br>5. 5 to 6 standard drinks<br>6. 3 to 4 standard drinks<br>7. 1 to 2 standard drinks

Table S.2 Cell sizes for fixed-effects models

	Women	Men
Continuously married	58968	59388
Married and >3 years prior to separation	2735	2319
-3 to -1 years prior to separation	1753	1486
Year of separation	657	541
1 to 2 years after separation	1123	925
3 to 4	925	781
5 to 6 years	777	618
7 to 8 years	655	509
>8 years	1400	1179

Notes: Data are from the Household, Income and Labour Dynamics in Australia (HILDA) survey

Table S.3 Sample description (fixed-effects sample)

	Women				Men			
	Marriage sample (control)		Separation sample (treated)		Marriage sample (control)		Separation sample (treated)	
	Mean	SD	Mean	SD	Mean	SD	Mean	SD
Regular drinking	0.53		0.54		0.72		0.71	
Binge drinking	0.05		0.09		0.06		0.11	
Any smoking (at all)	0.09		0.21		0.13		0.26	
Daily smoking	0.08		0.18		0.10		0.22	
Age	49.50	14.63	44.11	11.39	52.86	15.36	46.66	11.51
Relationship status								
Married	1.00		0.50		1.00		0.55	
Cohabiting	0.00		0.09		0.00		0.10	
Separated	0.00		0.20		0.00		0.19	
Divorced	0.00		0.21		0.00		0.16	
Nr of own resident children	1.11	1.21	1.33	1.16	1.04	1.19	0.85	1.06
Nr of own non-resident children	1.05	1.38	0.80	1.22	1.18	1.45	1.20	1.36
Labor force status								
Non-employed	0.39		0.30		0.29		0.18	
Employed	0.61		0.70		0.71		0.82	
Education								
Year 12 or below	0.41		0.40		0.29		0.32	
Diploma or certificate	0.26		0.31		0.41		0.42	
University	0.33		0.29		0.30		0.25	
Individual annual income (log)	6.45	5.21	7.65	4.80	7.39	5.36	8.55	4.81
ATSI								
Not of indigenous origin	0.99		0.98		0.99		0.98	
Indigenous origin	0.01		0.02		0.01		0.02	
First language learned at home								
English	0.46		0.54		0.54		0.61	
Not English	0.54		0.46		0.46		0.39	
Country of birth								
Australia	0.75		0.80		0.73		0.79	
Not Australia	0.25		0.20		0.27		0.21	
N Observations	58,968		10,025		59,388		8,358	

Table S.4 Average likelihood of drinking and smoking by gender and across the marital dissolution process (fixed-effects sample)

	Regular drinking		Binge drinking		Any smoking		Daily smoking	
	Women	Men	Women	Men	Women	Men	Women	Men
**Separation Sample**								
Married, >4 years prior to separation	0.51	0.72	0.07	0.11	0.19	0.25	0.16	0.21
-3 to -1 years prior to separation	0.53	0.71	0.11	0.11	0.20	0.27	0.18	0.22
Year of separation	0.56	0.72	0.12	0.11	0.26	0.31	0.22	0.26
1 to 2 years after separation	0.58	0.70	0.11	0.12	0.23	0.30	0.19	0.24
3 to 4	0.59	0.70	0.11	0.12	0.23	0.29	0.19	0.25
5 to 6 years	0.54	0.69	0.08	0.10	0.22	0.26	0.18	0.22
7 to 8 years	0.52	0.69	0.09	0.11	0.24	0.22	0.19	0.19
More than 8 years	0.53	0.69	0.06	0.09	0.20	0.21	0.18	0.17
**Continuously Married Sample**								
Average across all panel waves	0.53	0.72	0.05	0.06	0.09	0.13	0.08	0.10
Time after panel entry:								
0-2 years after	0.49	0.70	0.05	0.06	0.12	0.17	0.10	0.14
3-5 years after	0.51	0.72	0.04	0.06	0.11	0.15	0.09	0.12
6-8 years after	0.53	0.72	0.05	0.05	0.10	0.13	0.08	0.11
9-11 years after	0.53	0.72	0.05	0.06	0.09	0.12	0.07	0.10
12-14 years after	0.53	0.72	0.05	0.06	0.08	0.11	0.07	0.09
15-17 years after	0.54	0.72	0.05	0.06	0.07	0.11	0.06	0.09
18-21 years after	0.54	0.72	0.05	0.06	0.06	0.09	0.05	0.07

Table S.5. Fixed-effects linear probability models of marital dissolution and health behaviors over the pooled sample and disaggregated by gender.

	Any smoking			Daily smoking			Regular drinking			Binge drinking		
	Pooled B/(SE)	Women B/(SE)	Men B/(SE)	Pooled B/(SE)	Women B/(SE)	Men B/(SE)	Pooled B/(SE)	Women B/(SE)	Men B/(SE)	Pooled B/(SE)	Women B/(SE)	Men B/(SE)
Dissolution (ref.: married, >4 years prior separation)												
-3 to -1 years prior to separation	0.02*	0.01	0.02	0.01	0.01	0.01	0.02*	0.03*	0.02	0.02**	0.02*	0.01
	(0.01)	(0.01)	(0.01)	(0.01)	(0.01)	(0.01)	(0.01)	(0.01)	(0.01)	(0.01)	(0.01)	(0.01)
Year of separation	0.06***	0.06***	0.05***	0.05***	0.06***	0.04**	0.05***	0.08***	0.02	0.02*	0.03*	0.01
	(0.01)	(0.01)	(0.02)	(0.01)	(0.01)	(0.02)	(0.01)	(0.02)	(0.02)	(0.01)	(0.01)	(0.01)
1 to 2 years after separation	0.05***	0.05***	0.05**	0.03***	0.04***	0.03	0.04**	0.07***	-0.00	0.02*	0.02	0.03
	(0.01)	(0.01)	(0.02)	(0.01)	(0.01)	(0.02)	(0.01)	(0.02)	(0.02)	(0.01)	(0.01)	(0.02)
3 to 4	0.05***	0.05**	0.05*	0.04**	0.04**	0.03	0.03*	0.07**	-0.01	0.02	0.00	0.03
	(0.01)	(0.02)	(0.02)	(0.01)	(0.02)	(0.02)	(0.02)	(0.02)	(0.02)	(0.01)	(0.01)	(0.02)
5 to 6 years	0.04**	0.04*	0.04	0.03*	0.04*	0.01	0.01	0.03	-0.02	-0.00	-0.01	0.01
	(0.01)	(0.02)	(0.02)	(0.01)	(0.02)	(0.02)	(0.02)	(0.02)	(0.02)	(0.01)	(0.02)	(0.02)
7 to 8 years	0.04*	0.05**	0.01	0.02	0.04*	-0.01	-0.01	0.01	-0.02	0.00	-0.01	0.01
	(0.02)	(0.02)	(0.02)	(0.01)	(0.02)	(0.02)	(0.02)	(0.03)	(0.03)	(0.01)	(0.02)	(0.02)
More than 8 years	0.01	0.03	-0.01	0.00	0.03	-0.03	-0.01	0.00	-0.03	-0.01	-0.03	0.01
	(0.02)	(0.02)	(0.03)	(0.02)	(0.02)	(0.03)	(0.02)	(0.03)	(0.03)	(0.01)	(0.02)	(0.02)
N Observations	136739	68993	67746	136739	68993	67746	136739	68993	67746	136739	68993	67746
N Individuals	13296	6607	6689	13296	6607	6689	13296	6607	6689	13296	6607	6689

* p<.05, ** p<.01, *** p<.001; All models account for age, dummies for each year, and year at divorce

Table S.6 Predicted probabilities in the reference year for models presented in Table S.4

	Pooled	Women	Men
Any smoking	0.12	0.10	0.14
Daily smoking	0.10	0.09	0.12
Regular drinking	0.57	0.52	0.72
Binge drinking	0.06	0.05	0.06

Table S.7. Fixed-effects linear probability models of marital dissolution and smoking by gender and education

	Any smoking				Daily smoking			
	Women: Below university degree	Women: University degree	Men: Below university degree	Men: University degree	Women: Below university degree	Women: University degree	Men: Below university degree	Men: University degree
	B/(SE)	B/(SE)	B/(SE)	B/(SE)	B/(SE)	B/(SE)	B/(SE)	B/(SE)
Marital dissolution (ref.: married, >4 years prior dissolution)								
-3 to -1 years prior to separation	0.02	0.00	0.03*	0.00	0.01	0.01	0.02	-0.02
	(0.01)	(0.01)	(0.01)	(0.02)	(0.01)	(0.01)	(0.01)	(0.02)
Year of separation	0.07***	0.05*	0.07***	0.01	0.06***	0.04*	0.05*	0.03
	(0.02)	(0.02)	(0.02)	(0.02)	(0.02)	(0.02)	(0.02)	(0.02)
1 to 2 years after separation	0.05**	0.05*	0.07***	0.01	0.05**	0.03*	0.04	0.01
	(0.02)	(0.02)	(0.02)	(0.03)	(0.02)	(0.01)	(0.02)	(0.03)
3 to 4	0.06**	0.05	0.07**	0.01	0.05*	0.04	0.06*	-0.02
	(0.02)	(0.03)	(0.03)	(0.03)	(0.02)	(0.03)	(0.02)	(0.03)
5 to 6 years	0.05*	0.05	0.05*	0.03	0.05*	0.02	0.03	-0.01
	(0.02)	(0.03)	(0.03)	(0.04)	(0.02)	(0.03)	(0.03)	(0.04)
7 to 8 years	0.06**	0.06*	0.02	0.04	0.04*	0.05	-0.00	-0.02
	(0.02)	(0.03)	(0.03)	(0.03)	(0.02)	(0.03)	(0.03)	(0.04)
More than 8 years	0.03	0.04	0.01	-0.03	0.03	0.04	-0.02	-0.04
	(0.03)	(0.03)	(0.03)	(0.03)	(0.03)	(0.03)	(0.03)	(0.04)
Accounted for age, year dummies, divorce	Yes	Yes	Yes	Yes	Yes	Yes	Yes	Yes
N Observations	45671	23293	47296	20406	45671	23293	47296	20406
N Individuals	4362	2242	4743	1943	4362	2242	4743	1943

* p<.05, ** p<.01, *** p<.001

Table S.8. Fixed-effects linear probability models of marital dissolution and drinking by gender and education

	Regular drinking				Binge drinking			
	Women: Below university degree	Women: University degree	Men: Below university degree	Men: University degree	Women: Below university degree	Women: University degree	Men: Below university degree	Men: University degree
	B/(SE)	B/(SE)	B/(SE)	B/(SE)	B/(SE)	B/(SE)	B/(SE)	B/(SE)
Marital dissolution (ref.: married, >4 years prior dissolution)								
-3 to -1 years prior to separation	0.01	0.02	0.03*	-0.02	0.03*	0.01	0.01	0.03
	(0.01)	(0.03)	(0.02)	(0.02)	(0.01)	(0.01)	(0.01)	(0.01)
Year of separation	0.05*	0.06	0.07**	-0.01	0.03*	0.02	0.02	0.02
	(0.02)	(0.04)	(0.02)	(0.03)	(0.02)	(0.02)	(0.02)	(0.03)
1 to 2 years after separation	0.04	0.08*	0.03	-0.01	0.02	0.01	0.02	0.04
	(0.02)	(0.04)	(0.02)	(0.04)	(0.02)	(0.01)	(0.02)	(0.03)
3 to 4	0.02	0.07	0.03	-0.00	0.00	0.01	0.03	0.04
	(0.03)	(0.04)	(0.03)	(0.04)	(0.02)	(0.02)	(0.02)	(0.04)
5 to 6 years	0.02	0.04	0.02	-0.04	-0.01	0.00	0.01	0.04
	(0.03)	(0.05)	(0.03)	(0.05)	(0.02)	(0.02)	(0.02)	(0.05)
7 to 8 years	-0.01	0.01	0.05	-0.06	-0.01	-0.00	0.01	0.04
	(0.03)	(0.05)	(0.03)	(0.05)	(0.02)	(0.02)	(0.03)	(0.05)
More than 8 years	-0.02	0.01	0.03	-0.00	-0.04	0.01	0.01	0.02
	(0.03)	(0.05)	(0.03)	(0.06)	(0.02)	(0.02)	(0.03)	(0.05)
Accounted for age, year dummies, divorce	Yes	Yes	Yes	Yes	Yes	Yes	Yes	Yes
N Observations	45671	23293	47296	20406	45671	23293	47296	20406
N Individuals	4362	2242	4743	1943	4362	2242	4743	1943

* p<.05, ** p<.01, *** p<.001

Table S.9. Fixed-effects linear probability models of marital dissolution and smoking by gender and the presence of children

	Any smoking				Daily smoking			
	Women: No children present B/(SE)	Women: Children present B/(SE)	Men: No children present B/(SE)	Men: Children present B/(SE)	Women: No children present B/(SE)	Women: Children present B/(SE)	Men: No children present B/(SE)	Men: Children present B/(SE)
Marital dissolution (ref.: married, >4 years prior dissolution)								
-3 to -1 years prior to separation	0.00	0.02	0.04	0.02	0.01	0.01	0.02	0.02
	(0.02)	(0.01)	(0.02)	(0.02)	(0.01)	(0.01)	(0.02)	(0.02)
Year of separation	0.01	0.08***	0.02	0.07**	0.02	0.07***	0.02	0.07**
	(0.02)	(0.02)	(0.03)	(0.02)	(0.02)	(0.02)	(0.03)	(0.02)
1 to 2 years after separation	0.01	0.07**	0.04	0.06*	-0.00	0.06**	0.01	0.05*
	(0.02)	(0.02)	(0.04)	(0.03)	(0.02)	(0.02)	(0.03)	(0.03)
3 to 4	-0.01	0.07*	0.04	0.06	-0.03	0.06*	0.02	0.07
	(0.02)	(0.03)	(0.04)	(0.03)	(0.02)	(0.03)	(0.04)	(0.03)
5 to 6 years	-0.00	0.06*	0.05	0.04	-0.03	0.06*	0.01	0.05
	(0.02)	(0.03)	(0.04)	(0.04)	(0.02)	(0.03)	(0.04)	(0.04)
7 to 8 years	0.02	0.07*	0.02	0.01	-0.03	0.06	-0.01	0.03
	(0.03)	(0.03)	(0.04)	(0.04)	(0.03)	(0.03)	(0.04)	(0.04)
More than 8 years	-0.02	0.05	-0.02	0.01	-0.05	0.05	-0.03	0.03
	(0.04)	(0.04)	(0.05)	(0.05)	(0.04)	(0.04)	(0.05)	(0.05)
Accounted for age, year dummies, divorce	Yes	Yes	Yes	Yes	Yes	Yes	Yes	Yes
N Observations	61160	7833	61685	6061	61160	7833	61685	6061
N Individuals	6036	571	6252	437	6036	571	6252	437

* p<.05, ** p<.01, *** p<.001

Table S.10. Fixed-effects linear probability models of marital dissolution and drinking by gender and the presence of children

	Regular drinking				Binge drinking			
	Women: No children present B/(SE)	Women: Children present B/(SE)	Men: No children present B/(SE)	Men: Children present B/(SE)	Women: No children present B/(SE)	Women: Children present B/(SE)	Men: No children present B/(SE)	Men: Children present B/(SE)
Marital dissolution (ref.: married, >4 years prior dissolution)								
-3 to -1 years prior to separation	0.02	0.03	-0.00	0.02	0.01	0.03*	0.01	0.01
	(0.03)	(0.02)	(0.03)	(0.02)	(0.02)	(0.01)	(0.01)	(0.02)
Year of separation	-0.00	0.09**	0.03	0.04	0.01	0.04*	0.04	-0.01
	(0.03)	(0.03)	(0.03)	(0.03)	(0.02)	(0.02)	(0.02)	(0.03)
1 to 2 years after separation	0.00	0.09**	-0.01	0.01	-0.03	0.04	0.01	0.02
	(0.04)	(0.03)	(0.04)	(0.03)	(0.02)	(0.02)	(0.02)	(0.03)
3 to 4	-0.03	0.08*	-0.03	0.03	-0.07*	0.03	0.03	0.01
	(0.05)	(0.04)	(0.05)	(0.04)	(0.03)	(0.02)	(0.03)	(0.04)
5 to 6 years	-0.03	0.07	-0.02	0.00	-0.05	0.00	-0.00	-0.01
	(0.05)	(0.04)	(0.05)	(0.04)	(0.03)	(0.02)	(0.03)	(0.04)
7 to 8 years	-0.07	0.05	-0.04	0.03	-0.09**	0.02	-0.02	-0.00
	(0.05)	(0.05)	(0.06)	(0.05)	(0.03)	(0.03)	(0.03)	(0.05)
More than 8 years	-0.09	0.06	-0.08	0.05	-0.08**	-0.00	-0.00	-0.02
	(0.05)	(0.06)	(0.05)	(0.06)	(0.03)	(0.03)	(0.04)	(0.05)
Accounted for age, year dummies, divorce	Yes	Yes	Yes	Yes	Yes	Yes	Yes	Yes
N Observations	61160	7833	61685	6061	61160	7833	61685	6061
N Individuals	6036	571	6252	437	6036	571	6252	437

* p<.05, ** p<.01, *** p<.001

Table S.11. Cox models for smoking cessation.

	Model 1	Model 2	Model 3	Model 4	Robustness check
	HR/(SE)	HR/(SE)	HR/(SE)	HR/(SE)	HR/(SE)
Female	1.19	1.09	1.02	1.02	1.07
	(0.16)	(0.15)	(0.14)	(0.14)	(0.15)
Age at separation		0.97***	0.97***	0.97***	0.98**
		(0.01)	(0.01)	(0.01)	(0.01)
University degree			1.85***	1.86***	1.82***
			(0.30)	(0.30)	(0.30)
Living with children before separation				1.04	1.11
				(0.18)	(0.19)
Re-partner					1.37*
					(0.20)
Person Years	2615	2615	2615	2615	2615

Exponentiated coefficients (hazard ratios)
* p<.05, ** p<.01, *** p<.001

Table S.12. Cox models for regular drinking cessation.

	Model 1	Model 2	Model 3	Model 4	Robustness check
	HR/(SE)	HR/(SE)	HR/(SE)	HR/(SE)	HR/(SE)
Female	1.51***	1.43***	1.49***	1.51***	1.48***
	(0.16)	(0.15)	(0.16)	(0.16)	(0.16)
Age at separation		0.98***	0.98**	0.98**	0.98***
		(0.01)	(0.01)	(0.01)	(0.01)
University degree			0.58***	0.58***	0.59***
			(0.07)	(0.07)	(0.07)
Living with children before separation				0.88	0.87
				(0.11)	(0.11)
Re-partner					0.87
					(0.10)
Person Years	5835	5835	5835	5835	5835

Exponentiated coefficients (hazard ratios)
* p<.05, ** p<.01, *** p<.001

Figure S.1. Sample selection process.

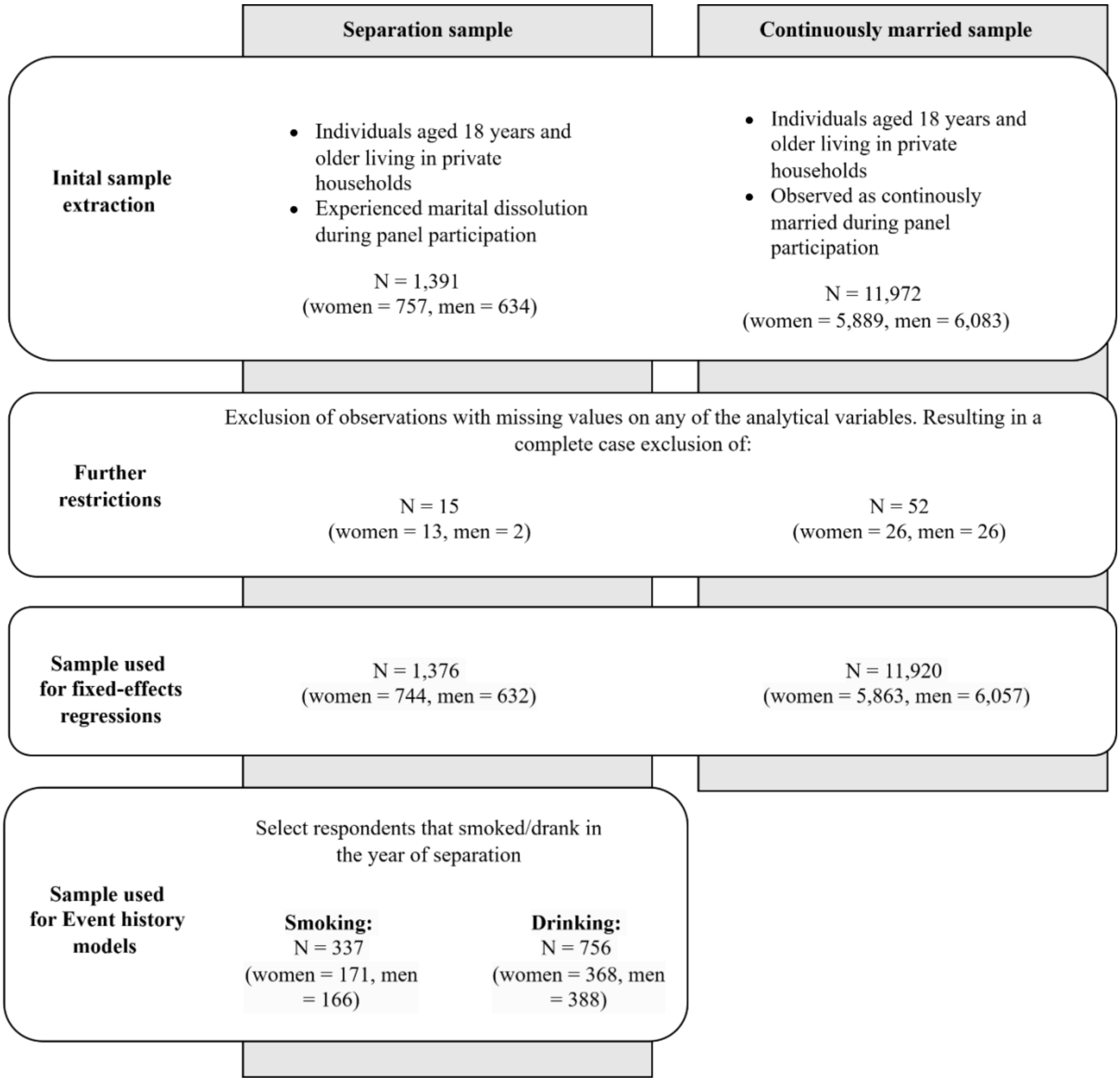

Figure S.2 Fixed-effects linear probability models for smoking and drinking controlling for our main covariates and re-partner.

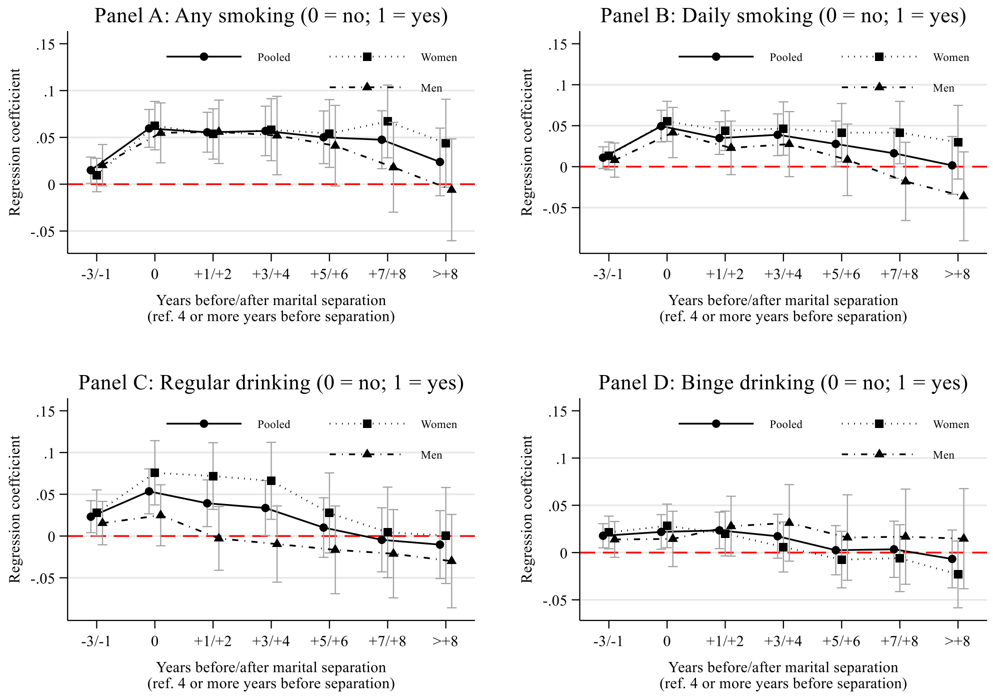

Notes: Whiskers indicate 95% confidence intervals. Data are from the HILDA survey (release 22, years 2001-2023).

Figure S.3 Fixed-effects linear probability models for smoking by education controlling for our main covariates and re-partner

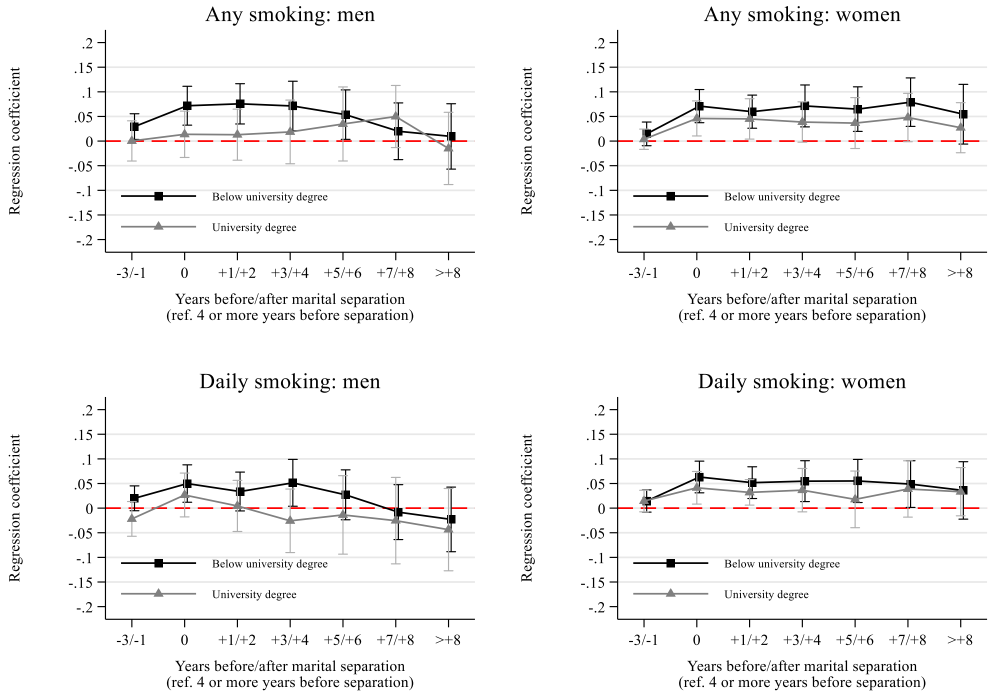

Notes: Whiskers indicate 95% confidence intervals. Data are from HILDA survey (release 22, years 2001-2023).

Figure S.4 Fixed-effects linear probability models for drinking by education controlling for our main covariates and re-partner

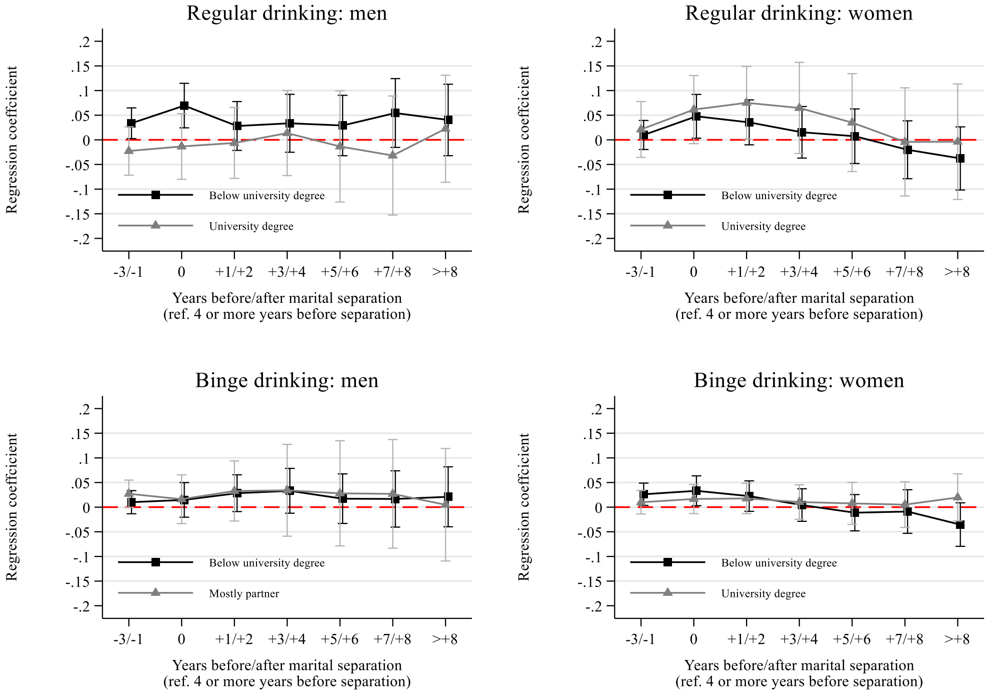

Notes: Whiskers indicate 95% confidence intervals. Data are from the HILDA survey (release 22, years 2001-2023).

Figure S.5 Fixed-effects linear probability models for smoking by presence of children before separation controlling for our main covariates and re-partner

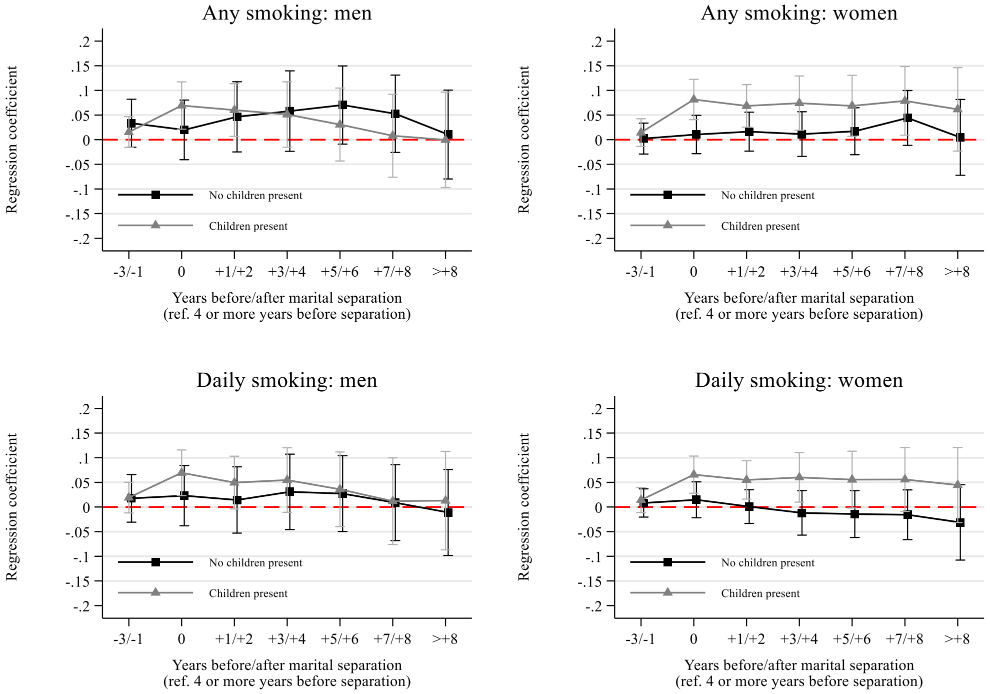

Notes: Whiskers indicate 95% confidence intervals. Data are from the HILDA survey (release 22, years 2001-2023).

Figure S.6 Fixed-effects linear probability models for drinking by presence of children before separation controlling for our main covariates and re-partner

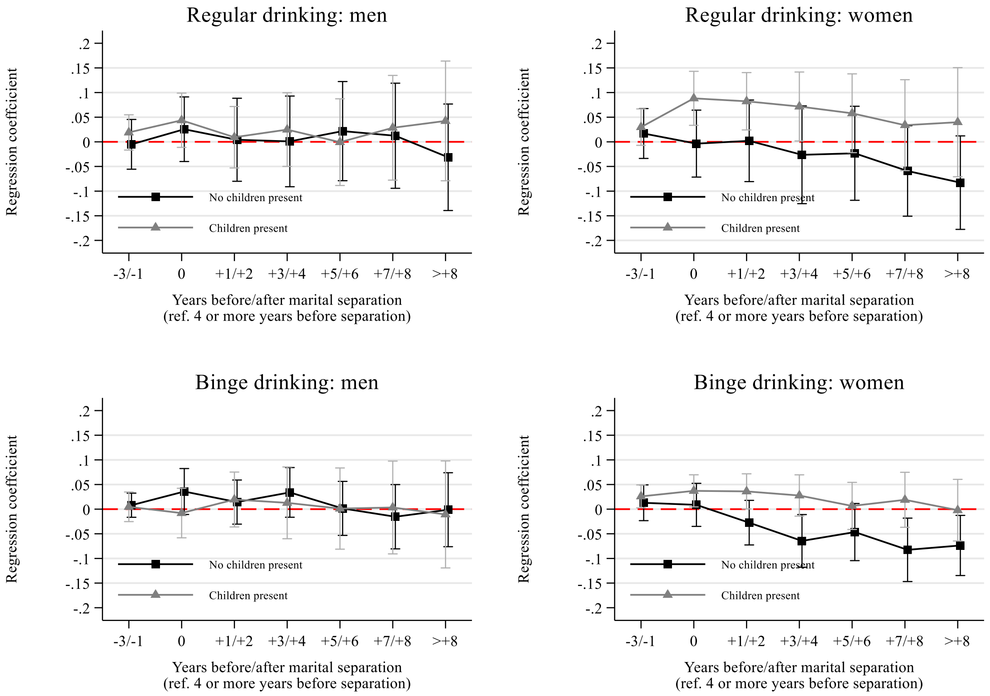

Notes: Whiskers indicate 95% confidence intervals. Data are from the HILDA survey (release 22, years 2001-2023).
